# Effect of misclassification of antiretroviral treatment status on the prevalence of transmitted HIV-1 drug resistance

**DOI:** 10.1186/1471-2288-12-30

**Published:** 2012-03-14

**Authors:** Hannah Castro, Deenan Pillay, Caroline Sabin, David T Dunn

**Affiliations:** 1 Medical Research Council Clinical Trials Unit, London, UK; 2 University College London (UCL)/Medical Research Council Centre for Medical Molecular Virology, UCL Medical School, London, UK; 3 UCL Medical School, London, UK

## Abstract

**Background:**

Estimates of the prevalence of transmitted HIV drug resistance (TDR) in a population are derived from resistance tests performed on samples from patients thought to be naïve to antiretroviral treatment (ART). Much of the debate over reliability of estimates of the prevalence of TDR has focused on whether the sample population is representative. However estimates of the prevalence of TDR will also be distorted if some ART-experienced patients are misclassified as ART-naïve.

**Methods:**

The impact of misclassification bias on the rate of TDR was examined. We developed methods to obtain adjusted estimates of the prevalence of TDR for different misclassification rates, and conducted sensitivity analyses of trends in the prevalence of TDR over time using data from the UK HIV Drug Resistance Database. Logistic regression was used to examine trends in the prevalence of TDR over time.

**Results:**

The observed rate of TDR was higher than true TDR when misclassification was present and increased as the proportion of misclassification increased. As the number of naïve patients with a resistance test relative to the number of experienced patients with a test increased, the difference between true and observed TDR decreased. The observed prevalence of TDR in the UK reached a peak of 11.3% in 2002 (odds of TDR increased by 1.10 (95% CI 1.02, 1.19, p(linear trend) = 0.02) per year 1997-2002) before decreasing to 7.0% in 2007 (odds of TDR decreased by 0.90 (95% CI 0.87, 0.94, p(linear trend) < 0.001) per year 2002-2007. Trends in adjusted TDR were altered as the misclassification rate increased; the significant downward trend between 2002-2007 was lost when the misclassification increased to over 4%.

**Conclusion:**

The effect of misclassification of ART on estimates of the prevalence of TDR may be appreciable, and depends on the number of naïve tests relative to the number of experienced tests. Researchers can examine the effect of ART misclassification on their estimates of the prevalence of TDR if such a bias is suspected.

## Background

Estimates of the prevalence of transmitted HIV drug resistance (TDR) in a population are derived from resistance tests performed on samples from patients thought to be naïve to antiretroviral treatment (ART). The certainty of whether a patient is naïve to treatment at the time a sample for resistance testing is taken relies on sources of information about a patient's treatment status. Patients who have moved countries or clinical centres may not remember the exact date of starting ART or choose not to share information about previous therapy with their current health care provider. Poor data collection methods and/or ways of recording data on clinical databases may also result in incomplete or incorrect information in an analysis, especially if databases from different clinical centres need to be linked to obtain a patient's full treatment history.

Much of the debate over reliability of estimates of the prevalence of TDR has focused on whether the sample population is representative [1]. However estimates of the prevalence of TDR will also be distorted if some ART-experienced patients are misclassified as ART-naïve, as has been discussed with reference to UK estimates [2,3]. As large, multicentre cohort studies are increasingly utilised to assess the burden of TDR on a national or regional level, the potential biases in such data must be addressed.

In this paper we quantify the potential extent of this bias and its impact on estimates of the prevalence of TDR, illustrate methods to obtain adjusted estimates of the prevalence of TDR under different assumptions of the rate of misclassification, and conduct sensitivity analyses of trends in the prevalence of TDR over time using data from the UK HIV Drug Resistance Database.

## Methods

The prevalence of TDR is calculated as the proportion of samples, from patients who are thought to be naïve to ART, with resistance (usually defined as 1 or more major resistance mutations from a published mutation list). In terms of treatment misclassification, and assuming truly ART-naïve patients are never misclassified as ART-experienced, and that rate of resistance in experienced patients who are misclassified as naïve is the same as the rate of resistance in experienced patients who are not misclassified, the observed rate of TDR is:

(1) $$Observed\;TDR\;\left({\hat{R}}_{N}\right)=\frac{R_{E}a+R_{N}B}{a+B}=\frac{R_{E}m+R_{N}T}{m+T}$$

where $m=\frac{a}{A}$ is the proportion of truly experienced patients with a resistance test misclassified as naïve (*a *is the number of truly experienced patients with a resistance test misclassified as naïve, *A *is the number of truly experienced patients with a resistance test), $T=\frac{B}{A}$, the ratio of the number of truly naïve patients with a resistance test (*B*) to the number of truly experienced patients with a resistance test, and *R_E _*(*E *= experienced) and *R_N _*(*N *= naïve) are the rate of resistance in truly experienced and truly naive patients with a resistance test respectively.

To examine the effect of misclassification of treatment status on the trend in the prevalence of TDR over time in the UK, the first resistance test per patient conducted whilst observed as naïve to ART (n = 18,577) and the last test per patient conducted after being observed as treatment experienced (n = 10,792) were identified from UK HIV Drug Resistance database (to end of 2007). The misclassification rate, *m*, was chosen to be 2%, 5% or 6%. The UK HIV Drug Resistance Database is a central repository of genotypic resistance tests carried out as part of routine clinical care in the United Kingdom [2]. A patient's observed treatment status is classified from demographic and clinical information obtained and linked from various sources; electronic data are provided by the UK Collaborative HIV Cohort study (UK CHIC) [4], the UK Register of HIV seroconverters [5], the Survey of Prevalent HIV Infections Diagnosed (SOPHID) [6], the HIV and AIDS New Diagnoses national database (HAP), and other hospital databases. Information on exposure to ART is also asked for on request forms for resistance tests, and is cross-checked with data from the electronic databases before classifying a patient's observed treatment status at the time of a resistance test; a patient's observed treatment status is recorded as unknown if there is conflicting information. The study has UK Multicentre Ethics Committee approval (MREC/01/2/10).

Major resistance mutations were identified using the 2009 IAS-USA guidelines [7]. Logistic regression was used to examine trends in the prevalence of TDR over time.

## Results

### Bias in the estimation of the prevalence of TDR under different assumptions of the misclassification rate

**Figure 1:**
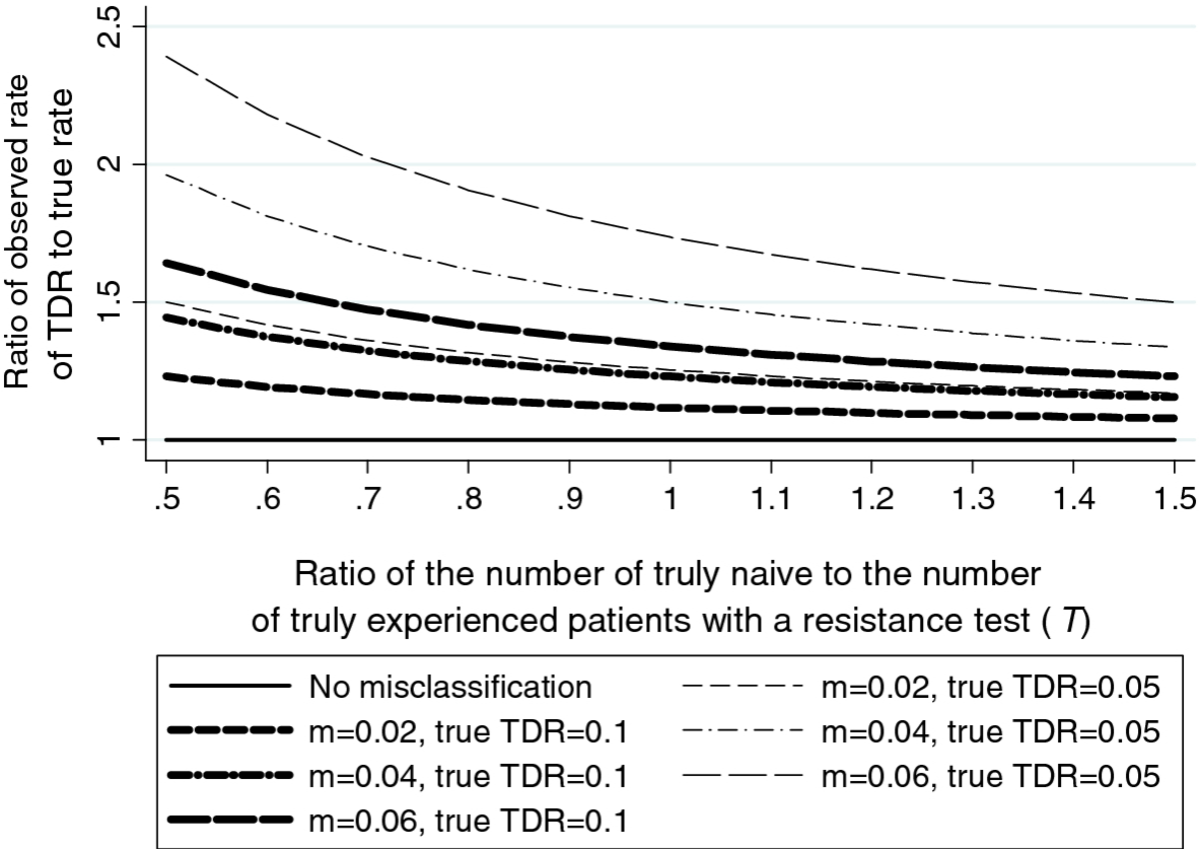
**The prevalence of TDR under different assumptions of the misclassification rate**. Rate of resistance in truly experienced was 0.7. m = misclassification rate. TDR = transmitted HIV drug resistance.

Using equation (1) (see Methods), as expected, the observed rate of TDR was higher than the rate of TDR in truly naive patients (true TDR) when misclassification was present, and increased as the proportion of misclassification increased. As the number of ART-naïve patients with a resistance test relative to the number of ART-experienced patients with a test increased, the difference between the true and observed rate of TDR decreased. Figure 1 shows the ratio of the observed rate of TDR to the true rate for illustrative values: *R_E _*= 0.7, *R_N _*= 0.05 or 0.1 and *m *= 0.02, 0.04 or 0.06. With 6% misclassification, a true rate of TDR of 10%, and 2 ART-experienced patients with a resistance test to every 1 ART-naïve patient with a test (*T *= 0.5), the rate of observed TDR was approximately 1.6 times the rate of true TDR. If a lower rate of true TDR was assumed (Figure 1, *R_N _*= 0.05) or higher rate of resistance in truly experienced patients was assumed (data not shown), the relative effect of misclassification was more pronounced, and vice versa if a higher rate of true TDR or lower rate of resistance in truly experienced patients was assumed (data not shown).

### Adjusted estimates of the prevalence of TDR

In practice, the misclassification rate in a surveillance population is unknown and estimates of the prevalence of TDR are based on patients classified as ART-naïve. However, sensitivity analyses to obtain adjusted estimates of the prevalence of TDR under different assumptions of the misclassification rate may be of interest. It can be shown that adjusted TDR can be written as:

$$Adjusted\;TDR=\frac{y-m\left(x+y\right)}{Y-m\left(X+Y\right)}$$

where *Y *(= *B *+ *a*) and *X *(= *A *- *a*) are the number of observed ART-naive and ART-experienced patients with a resistance test respectively, and *y *(= *R_N_B *+ *R_E_a*) and *x *(= *R_E_A *- *R_E_a*) are the number of observed ART-naive and ART-experienced patients with resistance (see Additional file 1 for derivation).

### Empirical adjustment of the prevalence of TDR to account for the effect of misclassification of treatment status: the trend over time in the UK

**Figure 2:**
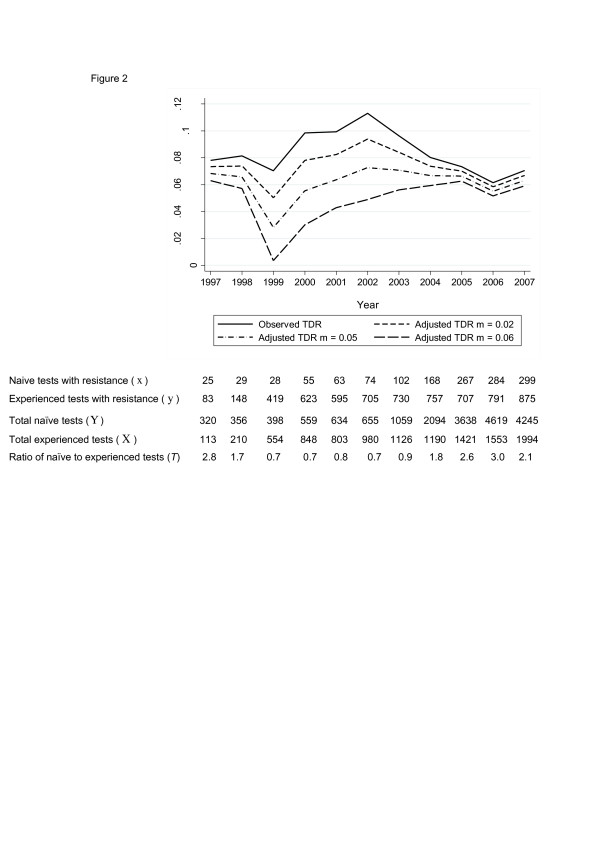
**Adjusted estimates of the prevalence of TDR in the UK over time**. TDR = transmitted HIV drug resistance.

The observed prevalence of TDR in the UK reached a peak of 11.3% in 2002 (odds of TDR increased by 1.10 (95% CI 1.02, 1.19, p(linear trend) = 0.02) per year 1997-2002) before decreasing to 7.0% in 2007 (odds of TDR decreased by 0.90 (95% CI 0.87, 0.94, p(linear trend) < 0.001) per year 2002-2007) (Figure 2). However, trends in adjusted estimates of the prevalence of TDR were different as the misclassification rate increased. When the ratio of the number of observed naïve tests to the number observed experienced tests was high (i.e. in 1997, 2005-2007), the effect of adjusting for misclassification was less than for years when the ratio was low (Figure 2). Adjusted TDR approached 0% in 1999 as the misclassification rate increased to 6%, due to the number of misclassified patients with resistance increasing to nearly equal the number of naïve patients with resistance.

The statistically significant upward linear trend between 1997 and 2002 seen for the observed prevalence of TDR was lost when the misclassification rate increased to just under 1% (data not shown). The statistically significant downward linear trend between 2002 and 2007 was lost when the misclassification rate increased to over 4% (data not shown).

## Discussion

We have shown that the effect of misclassification of antiretroviral treatment status on estimates of the prevalence of TDR may be appreciable, even if the rate of misclassification is low. The size of the effect depends on the number of naïve tests relative to the number of experienced tests, as well as the rate of resistance in naïve and experienced patients. If in a population resistance tests are being done more frequently in treatment naïve patients than in treatment experienced, the effect of misclassification of experienced patients as naïve on estimates of the prevalence of TDR will be diluted.

Estimating the misclassification rate in a population is challenging. Knowledge of the accuracy of clinical databases may suggest whether the misclassification rate in one setting may be higher or lower than in another setting but quantifying this difference is more difficult. When estimates of the prevalence of TDR in the UK were first obtained from the UK HIV Drug Resistance Database for all tests conducted in the UK up to 2003 [2] an exercise was carried out to try and verify the accuracy of the ART data. Hospital clinical notes or the resistance test request form (which contains information on exposure to antiretroviral therapy) were checked on a sample of supposedly ART-naive patients with resistant mutations against information from the databases mentioned in the Methods. Adjusting our findings from this exercise to account for the number of patients without resistance who may have been misclassified, we estimated an overall misclassification rate of approximately 2%.

In the UK the number of naïve tests conducted has increased over time more sharply than the number of experienced tests, despite the number of patients on ART increasing, and as a consequence the effect of misclassification of treatment status on estimates of the prevalence of TDR in recent years was minimal. The trend in the prevalence of TDR over time in the UK was dramatically altered when different rates of misclassification were assumed and the downward trend in the prevalence of TDR since 2002 was lost when the misclassification rate increased to over 4%. Whilst this implies that trends over time in the prevalence of TDR should be interpreted with caution, an estimated misclassification rate of approximately 2% provides reassurance that there has been a decline in the prevalence of TDR in the UK. This analysis assumed that the misclassification rate was constant over time, which may be incorrect if there are reasons to suspect that the reliability of treatment history has changed over time, for example, an increase in patients who have migrated from other countries and have an incomplete ART history.

Misclassification of treatment status is just one factor which may influence estimates of TDR, others include biased sampling [1], different definitions of transmitted resistance [8], the study of acute infections, chronic infections, or both [1], geographical differences [1], and the persistence of transmitted resistance mutations [9].

## Conclusion

The effect of misclassification of ART on estimates of the prevalence of TDR may be appreciable, and depends on the number of naïve tests relative to the number of experienced tests. Our simple formula for calculating adjusted prevalence of TDR allows researchers to examine the effect of ART misclassification on their estimates of the prevalence of TDR if such a bias is suspected. However the method presented depends on the rate of resistance in experienced patients and not all TDR surveillance studies may have this information available.

## Competing interests

The authors declare that they have no competing interests.

## Authors' contributions

HC and DTD designed the study. HC did the analysis and wrote the first draft of the manuscript. All authors made substantive comments on the different revisions of the manuscript, and read and approved the final manuscript.

## Pre-publication history

The pre-publication history for this paper can be accessed here:

http://www.biomedcentral.com/1471-2288/12/30/prepub

## Supplementary Material

Additional file 1 **Derivation of the formula for adjusted TDR**. Click here for file
