# Supplementary material for: Effect of misclassification of antiretroviral treatment status on the prevalence of transmitted HIV-1 drug resistance
Source: BMC Med Res Methodol. 2012 Mar 14;12:30. doi: 10.1186/1471-2288-12-30 (PMC3364874; doi:10.1186/1471-2288-12-30)
Supplement: Additional file 1 — Derivation of the formula for adjusted TDR. [file 1471-2288-12-30-S1.DOC]

**Derivation of the formula for adjusted TDR**

$$Y=B+a$$

$$X=A-a$$

$$y=R_{N}B+R_{E}a$$

$$x=R_{E}A-R_{E}a$$

$$m=\frac{a}{A}$$

*Adjusted TDR* = $\frac{y-m\left(x+y\right)}{Y-m\left(X+Y\right)}$

= $\frac{R_{N}B+R_{E}a-\frac{a}{A}\left(R_{E}A-R_{E}a+R_{N}B+R_{E}a\right)}{B+a-\frac{a}{A}\left(A-a+B-a\right)}$

= $\frac{AR_{N}B+AR_{E}a-aR_{E}A-aR_{N}B}{AB+Aa-aA-aB}$

= $\frac{BR_{N}\left(A-a\right)}{B\left(A-a\right)}$

= $R_{N}$
